# Life Cycle Assessment of an Integrated Membrane Treatment System of Anaerobic-Treated Palm Oil Mill Effluent (POME)

**DOI:** 10.3390/membranes12020246

**Published:** 2022-02-21

**Authors:** Khalisah Khairina Razman, Marlia M. Hanafiah, Abdul Wahab Mohammad, Ang Wei Lun

**Affiliations:** 1 Department of Earth Sciences and Environment, Faculty of Science and Technology, Universiti Kebangsaan Malaysia, UKM, Bangi 43600, Selangor, Malaysia; khalisahkhairina18@gmail.com; 2 Centre for Tropical Climate Change System, Institute of Climate Change, Universiti Kebangsaan Malaysia, UKM, Bangi 43600, Selangor, Malaysia; 3 Department of Chemical and Process Engineering, Universiti Kebangsaan Malaysia, UKM, Bangi 43600, Selangor, Malaysia; drawm@ukm.edu.my (A.W.M.); wl_ang@ukm.edu.my (A.W.L.); 4 Research Centre for Sustainable Process Technology (CESPRO), Faculty of Engineering and Built Environment, Universiti Kebangsaan Malaysia, Bangi 43600, Selangor, Malaysia

**Keywords:** life cycle assessment, integrated membrane, palm oil mill effluent, anaerobic digestion, anaerobic pre-treatment, adsorption, electro-oxidation, sustainability

## Abstract

A life cycle assessment of anaerobic-treated palm oil mill effluent (POME) was conducted to assess the environmental performance on two integrated treatment processes: the typical hollow fiber membrane ultrafiltration module coupled with adsorption and electro-oxidation as pretreatment. The analysis was undertaken using the ReCiPe 2016 method and SimaPro v9 software was employed using a ‘cradle-to-gate’ approach. The results showed that hollow fiber membrane from the adsorption integrated membrane impacted significantly at 42% to 99% across all impact categories for both processes. Overall, the electro-oxidation integrated membrane was discovered to have a lesser environmental impact, particularly on the ozone formation (human health) (HOFP) at 0.38 kg NOx-eq in comparison to the adsorption integrated membrane at 0.66 kg NOx-eq. The total characterization factor of the endpoint category for human health is 8.61 × 10^−4^ DALY (adsorption integrated membrane) and 8.45 × 10^−4^ DALY (electro-oxidation integrated membrane). As membrane treatment is closely linked to energy consumption, the environmental impact with different sources of energy was evaluated for both processes with the impacts decreasing in the following order: Grid > Biogas > Grid/Solar. Future research should concentrate on determining the overall ‘cradle-to-grave’ environmental impact of treating POME, as well as other scenarios involving membrane treatment energy utilization using LCA. This study can help decision-makers in identifying an environmentally sustainable POME treatment and management, especially in Malaysia.

## 1. Introduction

Malaysia has been one of the world’s largest producers and exporters of palm oil products. The number of palm oil mills is quickly expanding each year, increasing the capacity of fresh fruit bunch waste or effluent discharge. From 10 mills in 1960 to 410 mills in 2008, the number of palm oil mills has increased dramatically [1]. The waste formed during the processing of fresh fruit bunches (FFB) is known as palm oil mill effluent (POME), and it is the toughest waste to handle for mill operators [2]. This is because enormous amounts (in tons) are produced at a time. The use of resources in an unsustainable manner generates an imbalance in the natural environment. Agriculture, industry, residential use, and recreational activities all have a negative impact on our environment due to pollution and contamination [3]. Generally, wastewater from palm oil mills has a low pH as it contains organic acids with a high number of total solids, oil and grease and dissolved constituents such as protein, carbohydrate, nitrogenous compounds and lipids and minerals [4]. Water quality has become a critical issue, especially when it comes to determining how to produce a biologically acceptable water system [5]. The presence of these impurities results in difficulties in getting acceptable treated water which meets discharge regulations or reuse purposes.

POME is a mixture of lignocellulosic wastes, carbohydrates, and oil. POME has a very high chemical oxygen demand (COD) and biochemical oxygen demand (BOD), with COD levels frequently exceeding 80,000 mg/L [6]. When dumped into nearby rivers or lakes without treatment, palm oil mill effluent (POME) is a significant source of inland water pollution. To solve this issue, the ecosystem must be adequately and completely managed, and this can be done by treating the effluent to acceptable discharge limits set by local regulators before discharging it to the environment. Due to the high COD and BOD values, direct discharge of POME into the environment is discouraged, therefore, anaerobic digestion is used as the primary treatment process to lower waste volumes whilst producing beneficial byproducts. The treatment converts large organic molecules into mostly methane and carbon dioxide by bacteria without the presence of oxygen via an anaerobic digester. The small amounts of sludge formation, which reduces the challenges of biological sludge disposal, as well as the requirements for inorganic nutrients such as nitrogen and phosphorus, are the most significant advantages of anaerobic digestion [7]. Furthermore, the methane gas released is a highly valuable commodity that can be used for on-site energy consumption. There are various wastewater treatment methods utilising membrane systems such as using a membrane bioreactor [8,9], a membrane anaerobic system [10], an ultrafiltration membrane [11,12] and an integrated membrane [13]. However, as membrane fouling and efficiency in chemical/biological contaminant removal remain a challenge, there is a rising development of membrane process advancements to help improve selectivity and provide resistance towards fouling and energy consumption [14]. The adsorption process is a common example of this development predominantly using activated carbon as a tertiary treatment that eliminates the small amount of soluble organics, inorganic contaminants, and heavy metals. Identifying the specific characteristics of each wastewater plays a major role in designing an efficient treatment system [15]. This opinion is similar to [16], however, the study emphasized the importance of identifying the major foulant of the membrane processes from anaerobic effluents to develop the effective or required pretreatment. Regardless, these studies only concern themselves with the efficiency of the treatment and not the sustainability aspect of it to the environment. Taking into account the characteristics of POME, and in particular, the high concentrations of biodegradable organic compounds, a competitive technology for their neutralization may be anaerobic digestion [17]. Anaerobic treatment is a technologically sound and environmentally friendly method for the biodegradability of organic waste [18]. A well-implemented anaerobic digestion can reduce susceptibility to putrification, improve sanitation, reduce the volume of wastes and dry matter content, and lead to the production of high yields of CH_4_-rich biogas [19]. This has a positive effect on the further stages of POME purification, including membrane processes [20].

Global warming, water scarcity, and soil degradation are not just observable in rural areas, but also in metropolitan places where anthropogenic activities have an impact [21]. Rapid economic growth, swiftly changing lifestyles, and rural-urban migration are all contributing to this increasing trend [22]. To provide environmentally acceptable technologies for future development, the environmental burdens generated by the implementation of the integrated membrane must be examined. Hence, life cycle assessment (LCA) is a comprehensive tool to examine a given system or product’s environmental impacts at all phases of its life cycle, from ‘cradle-to-grave’ [23]. Nevertheless, it is possible to create various system limits for LCA studies, such as ‘cradle-to-gate’ studies that do not address product distribution and consumption, depending on the study’s goals and the availability of data and/or impact assessment methodologies [24].

There are only a small number of studies have been conducted on the long-term sustainability of these membrane processes using LCA, with the results demonstrating significant contamination removal based on existing parameters that comply with local regulations. When it comes to POME wastewater treatment, membrane technology is usually more concerned with the membrane’s efficiency in reclaiming the wastewater than with its environmental consequences and long-term sustainability.

From recent and related studies [25,26,27,28], it can be identified that these LCA of POME treatment studies only considered ‘gate-to-gate’ analysis and mostly took the midpoint approach. The system boundaries of the LCA studies were ‘gate-to-gate’ with the defined goals of analyzing the environmental impacts or sustainability of the POME treatment system. The studies mainly utilized SimaPro software with ReCiPe [27,28] and the Impact 2002+ methodology [25], apart from [26] which used GaBi software employing the CML 2001 method. All the studies had different FUs and chose the midpoint approach excluding [27]. In [27] it was found that employing a constant electric field of 300 V/c on a conductive membrane with a 1:9 optimum weight ratio with graphene oxide (GO)/multi-walled carbon nanotubes (MWCNTs) and a carbon nanomaterials concentration of 5% weight is environmentally feasible and efficient at reducing membrane fouling for electrically enhanced POME filtration. This is based on the functional unit (FU) of 1 L of permeate. As for [26], the paper noted that the two distinct POME treatment technologies contributed to a negative result signifying a net benefit to the environment, except for eutrophication potential (EP). The FU for this study was 1 kWh of electricity generated from POME.

Hence, motivated by the lack of LCA studies treating POME using a membrane system, the goal of this study is to assess the environmental performance of treating anaerobic POME using an integrated membrane from ‘cradle-to-gate’ with consideration of midpoint and endpoint approaches. A ‘cradle-to-gate’ system boundary was selected to allow a more holistic study to be conducted and due to the availability of data. Not only that, previous studies did not consider ‘cradle-to-gate’ as part of their evaluation, making it crucial to conduct LCA with such a system boundary. As a single membrane process is rarely feasible, the pretreatment process is included as this will help explore the environmental impacts of this integrated process. This includes the assessment of the raw material extraction and manufacturing process of the POME treatment that will be discussed in the following section. The findings could help improve the efficiency and long-term viability of POME membrane treatment technology as a green waste management technique and a treatment option.

## 2. Materials and Methods

The study follows the LCA framework according to the ISO 14040 [29] series based on the four phases of goal and scope definition, life cycle inventory, life cycle impact assessment and interpretation.

### 2.1. Goal and Scope Definition

**Figure 1:**
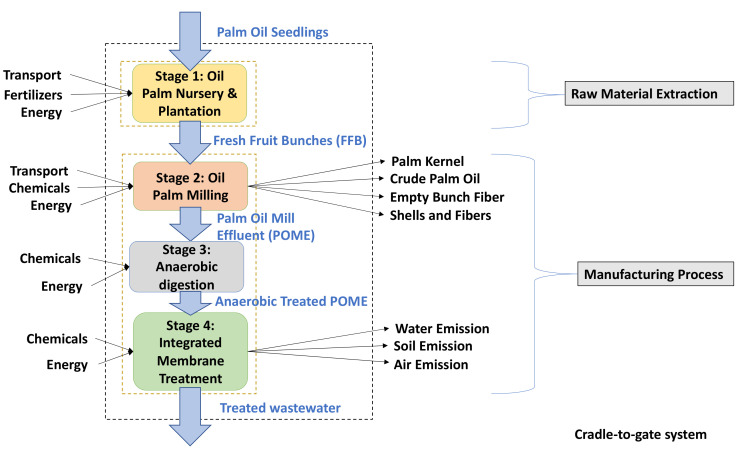
System boundary of the integrated membrane treatment system.

The goal of this study is to evaluate the environmental impact and environmental hotspots of an integrated membrane system in treating anaerobic palm oil mill effluent (POME) with several scenarios involving adsorption and electro-oxidation as pretreatment processes. The boundary of this study primarily focuses on ‘cradle-to-gate’ which considers the raw material extraction phase and the manufacturing phase of the treatment system. The raw material extraction encompasses the phase of palm oil nursery to palm oil plantation whilst the manufacturing process considers the palm oil milling, pretreatment of POME, and the integrated membrane treatment. Figure 1 represents the boundaries of the study in their respective labelled stages. Additionally, 1 m^3^ of treated wastewater is defined as the FU of this study which is used to assess the associated environmental impacts. As the study’s main goal is to identify the environmental hotspots and impacts of treating anaerobic POME using an integrated membrane, the membrane decommissioning and disposal phases are out of scope and were not considered.

### 2.2. Life Cycle Inventory

The most important aspect of the LCA is the data collection step, which is from the second phase of the life cycle inventory. For this study, the data collected were broken down into four stages as seen in Figure 1 from various sources including on-site data, relevant literature reviews and databases available in SimaPro v9. The sources of the secondary data selected in this study are divided into two, namely, Stage 1–3 and Stage 4. The key factor in selecting data for Stage 4 was choosing studies that defined their FU as 1 m^3^ of treated water [30,31,32] which is in par with this study’s FU since Stage 4 focuses on the treatment phase of the system. However, Stage 1–3 used a source that defined their study’s FU as 1 ton of POME [33] as it is focusing more on the palm oil plantation and milling to obtain POME. The selected studies were from various parts of the world with only [33] representing a dataset from Malaysia. Regardless, the background data used in EcoInvent 3.6 represented the global production except for energy consumption which is chosen to represent Malaysia’s electricity grid.

#### 2.2.1. Stage 1—Oil Palm Nursery and Plantation

The forest area in Malaysia has been transformed into plantation land, with peat making up most of the soil. Because it is commonly considered the most efficient carbon sink, agricultural peatland could contribute greatly to global warming [33]. A double-stage nursery system is the most typical nursery method in Malaysia. Pre-nursery and main nursery are the two primary stages of the double-stage nursery system [34]. For this stage, secondary data were obtained from [33] in which 8 young oil palms were produced from 15 germinated seeds grown in polybags. To provide nutrients to the seedlings, organic fertilizers were supplied, these are important for plant growth and development. Pesticides such as cypermethrin, dithane and glufosinate ammonium were also used to keep the seedlings safe from insects and infections. The inventory data for Stage 1 is presented in Table 1 and Table 2 with data sources from a relevant literature review.

#### 2.2.2. Stage 2—Oil Palm Milling

The palm oil mill converts fresh fruit bunch from the previous stage to crude palm oil and POME. The POME produced from this stage will then be treated using the two processes of adsorption and electro-oxidation of an integrated membrane in the following stage. Table 3 presents the data obtained by a relevant literature review for oil palm milling.

#### 2.2.3. Stage 3—Anaerobic Digestion

The pre-treatment stage has three major inputs namely electricity, water, and POME which allow anaerobic digestion to occur (Table 4). The secondary data was obtained from [33] for this stage. A total of 1 t of POME and 11 kg of processed water was supplied for the anaerobic digestion along with 0.0249 kWh of electricity; this produced 0.773 m^3^ of anaerobically digested POME. Most of the output to the environment for this stage is made up of biogas production, however this is out of the scope of this study and will not be considered.

#### 2.2.4. Stage 4—Integrated Membrane Treatment

**Figure 2:**
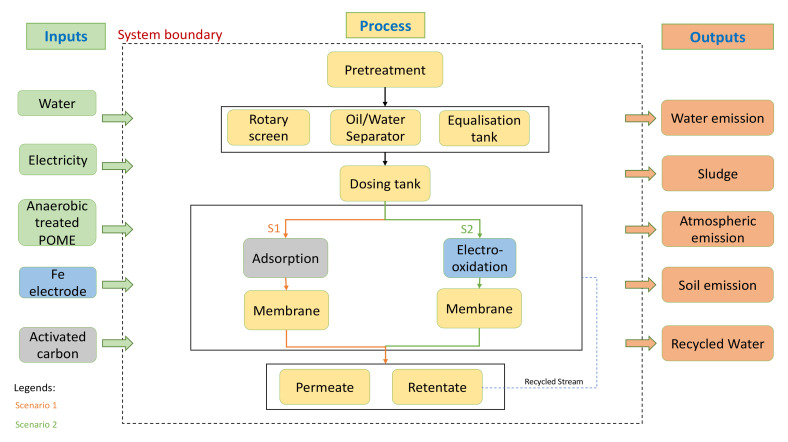
System boundary of Stage 4.

Figure 2 shows the system input-output of the adsorption and electro-oxidation integrated membrane treatment system with two different scenarios of treatment.

##### Integrated Adsorption-Membrane Process

The data sources used in the inventory analysis are summarized in Table 5. As can be seen, the material for the entire system is extracted from several data sources. The inventory data is further divided into three types of input, namely granular activated carbon (GAC) production, the membrane, and the membrane treatment itself. The material and energy needed to create and regenerate GAC production were determined using literature sourced from [30], whereas information regarding the material and energy needed for the membrane treatment was sourced from [31]. The membrane is made up of an ultrafiltration hollow fiber membrane with an area of 10 m^2^.

##### Integrated Electro-Oxidation Membrane Process

The inventory data that were used to implement the electro-oxidation unit for this study was provided by [32] (Table 6). About 3.55 kWh of energy was needed for this treatment stage. The unit consists of three sheltered reinforced concrete water storage tanks that are double-lined with epoxydic resin to prevent corrosion and equipped with 67 anode-cathode pairs [32]. The same type of hollow fiber membrane as the adsorption-integrated membrane process was utilized.

### 2.3. Life Cycle Impact Assessment

The inventory data were collected and analysed using SimaPro v9 software. SimaPro is an LCA tool that may be used to track the performance of a product or service’s sustainability. This software can methodically analyze a complex life cycle and assess the environmental impact of a product or service at each stage. The libraries selected as the background data for the software were EcoInvent 3.6, Agri-footprint 5.0, Methods, and USLCI.

**Figure 3:**
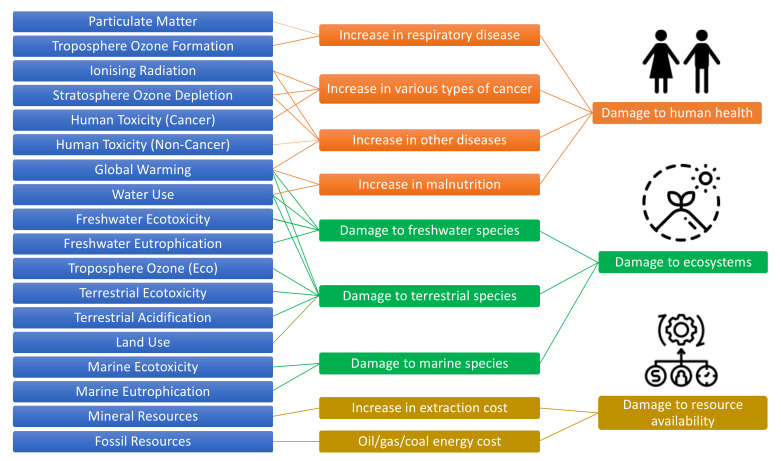
Overview of the midpoint and endpoint categories for the ReCiPe method [35].

Calculations were made based on the ReCiPe 2016 method developed by RIVM, Radboud University Nijmegen, Leiden University and PRé Sustainability. ReCiPe 2016 has the advantage of a more comprehensive collection of impact categories at the midpoint level [35]. Both midpoints and endpoints were taken into account to calculate the impact categories by using the ReCiPe 2016 (World-H) midpoint method and (World-H/H) endpoint method. At the midpoint level, ReCiPe 2016 assesses 18 different impact categories: global warming (GWP), stratospheric ozone depletion (ODP), ionizing radiation (IRP), ozone formation (human health) (HOFP), fine particulate matter formation (PMFP), ozone formation (terrestrial ecosystems) (EOFP), terrestrial acidification (TAP), freshwater eutrophication (FEP), marine eutrophication (MEP), terrestrial ecotoxicity (TETP), freshwater ecotoxicity (FETP), marine ecotoxicity (METP), human carcinogenic toxicity (HTPc), human non-carcinogenic toxicity (HTPnc), land use change (LUC), mineral resource scarcity (SOP), fossil resource scarcity (FFP), and water consumption (WCP) (Figure 3).

### 2.4. Interpretation of Scenario and Sensitivity Analysis

The outcomes are assessed by looking into the primary activities and chemical emissions that lead to each stage’s highest environmental burden. The following are the descriptions of each system: 

System 1: Treatment of anaerobic POME with an integrated membrane utilizing adsorption process.

System 2: Treatment of anaerobic POME with an integrated membrane utilizing electro-oxidation.

To determine the level of improvement in terms of environmental loads, a sensitivity analysis was conducted. Various components, including assumptions, data sources, characterization criteria, and data ranges, are modified in a sensitivity analysis. While this method adds to the complexity of LCA research, it also provides insight into the data and helps with the suggestions and decision-making process [24]. This is necessary to determine which stages and/or emissions contribute the most to the total environmental impact.

## 3. Results and Discussion

The results were analyzed according to their categories of midpoint and endpoint characterization factors. At the midpoint level, each unit represents their respective resource extracted or emissions released per kg. This can be seen from each stressor such as GWP with the unit of kg CO_2_-eq, ODP (kg CFC11-eq), and Ozone Formation (kg NOx-eq). As for the endpoint level, human health is represented by the unit DALY (disability-adjusted life years), ecosystem quality with PDF species⋅year, and resource availability with USD2013. Endpoint analysis may allow for more structured and informed weighting, particularly in terms of scientific aggregation across categories in terms of common parameters (for example, human health impacts associated with climate change can be compared to those associated with ozone depletion using a common metric like DALYs) [36]. The unit for ecosystem quality (PDF species⋅year) is defined by the local species loss when integrated over time. As for resource availability, the unit USD2013 indicated additional expenses associated with future mineral and fossil resource extractions [33].

### 3.1. Environmental Hotspots of Adsorption Integrated Membrane

#### 3.1.1. Midpoint Approach

**Figure 4:**
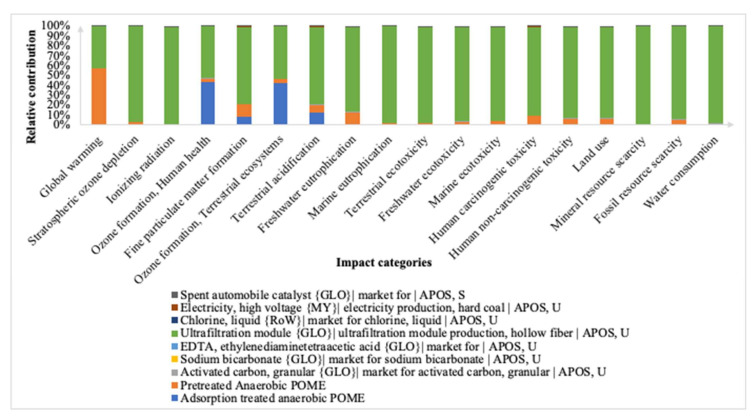
Impact categories at midpoint level for adsorption integrated membrane.

Figure 4 shows the midpoint impact categories for the adsorption integrated membrane and their relative contributions. The characterization factors from the system consist of both the anaerobic digestion stage (anaerobic POME) and the adsorption process (adsorption treated anaerobic POME, sodium bicarbonate, EDTA, activated carbon and spent catalyst). It can be observed that the major overall environmental hotspot is from the adsorption stage from the production of hollow fiber membrane contributing 42% to 99% of the total impact. The categories that were being impacted significantly by the hollow fiber membrane were mainly TETP, HTPnc and GWP. In [37] it was found that the type of solvent and polymer used as well as electricity source during the production of the hollow fiber membrane were key determinants of the environmental impact. Hence, these membrane manufactures can be made in an even more environmentally friendly manner by using electricity generated from renewable sources along with green solvents during production. The ultrafiltration hollow fiber membrane construction is generally assumed to be modelled using a batch process involving one polymer solution that serves as the foundation to calculate material and energy consumption per m^2^ of hollow fiber [38].

Besides that, the adsorption treated anaerobic POME significantly contributed towards HOFP, EOFP and TAP from 12% to 43% of the overall impact. The specific values in their respective units for all 18 characterization factors of the midpoint and endpoint categories are summarized in Tables S1 and S2. The total characterization value of GWP for the adsorption treatment of anaerobic POME was 583.87 kg CO_2_-eq, followed by TETP at 436.32 kg 1,4-DCB and HTPnc at 197.86 kg 1,4-DCB. The impact category contributing the least towards the characterization factors was the ODP with only 9.89 × 10^−5^ kg CFC_11_-eq from the materials of the adsorption process.

#### 3.1.2. Endpoint Approach

**Figure 5:**
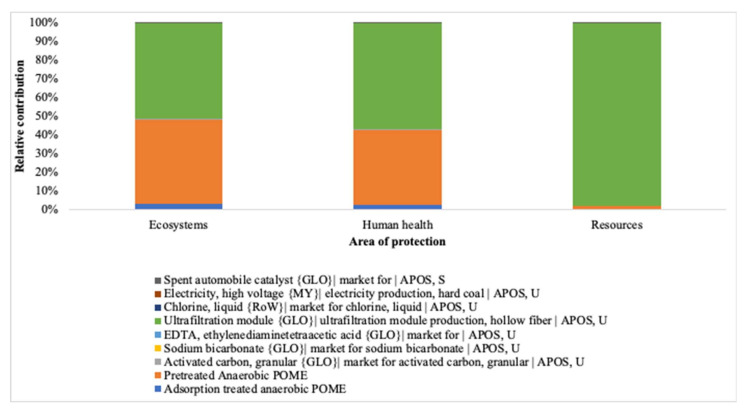
Impact categories at endpoint level for adsorption integrated membrane.

In terms of human health, ecosystem quality, and resource availability, the results demonstrated that adsorption and pretreatment dominated the environmental impacts (Figure 5). The adsorption-integrated membrane stage is a major contributor across all three endpoint categories, with the hollow fiber membrane making up most of it with up to 98% relative contribution compared to other materials used for the treatment which contributed less than 1%. In terms of the characterization factors in their areas of protection, the membrane impacted 4.86 × 10^−4^ DALY, 1.10 × 10^−6^ PDF species⋅year and 16.40 USD2013. The overall stages impacted the categories at 8.61 × 10^−4^ DALY, 2.14 × 10^−6^ PDF species⋅year and 16.83 USD2013. It can be noted that the membrane module affected the resources which may signify the high cost of the integrated membrane setup.

### 3.2. Environmental Hotspots of Electro-Oxidation Integrated Membrane

#### 3.2.1. Midpoint Approach

**Figure 6:**
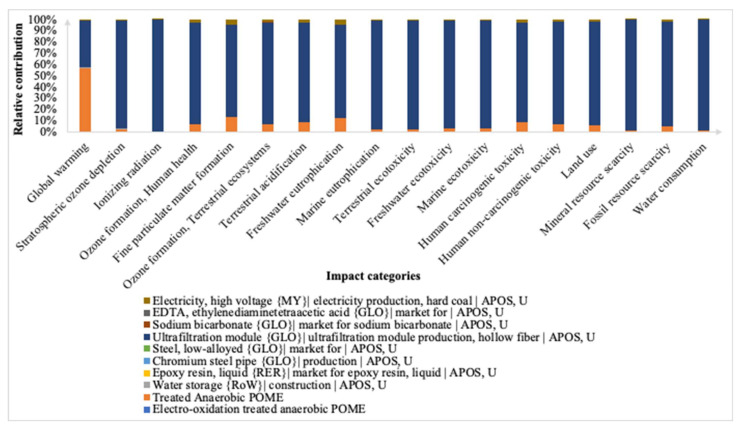
Impact categories at midpoint level for electro-oxidation integrated membrane.

The electro-oxidation integrated membrane midpoint impact categories are depicted in Figure 6 in their relative contributions. Across all 18 midpoint impact categories, the hollow fiber membrane used during electro-oxidation contributed majorly to the environmental impact of the integrated membrane treatment. The membrane module makes up 42% to 99% of their relative contribution from the total impact. The second highest impact originates from the anaerobic digestion stage of anaerobic POME, affecting GWP the most with 331.93 kg CO_2_-eq, with a relative contribution of 57% across the impact category. On the other hand, the materials used to treat the POME such as chromium steel pipe, epoxy resin and steel do not majorly affect the characterization factors. However, amongst the materials, steel contributes the highest with 0.05 kg 1,4-DCB of HTPnc, 8.02 × 10^−4^ kg Cu-eq of MRS, and 4.01 × 10^−3^ kg 1,4-DCB to FETP. The other counterparts contributed less than 1% to the same impact categories. Despite electrochemical processes having energy consumption as one of the known key challenges, electricity consumption was not found to be an environmental hotspot for this study. This could be because various factors, such as the anode and cathode material properties, applied current density or cell voltage, chloride concentration, and other operational parameters which might differ from other studies, could have affected the energy consumption [39].

#### 3.2.2. Endpoint Approach

**Figure 7:**
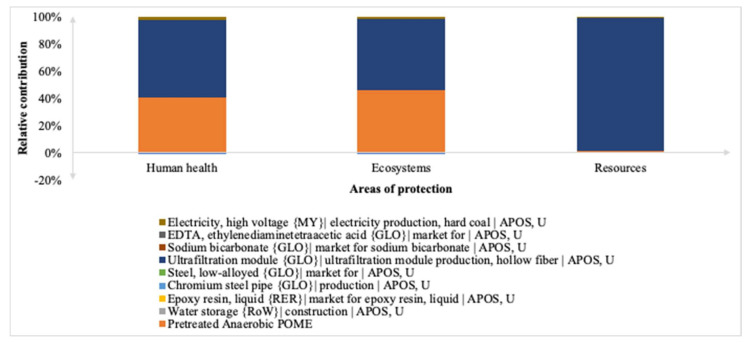
Impact categories at endpoint level for electro-oxidation integrated membrane.

When it comes to the endpoint level characterization factors, the electro-oxidation integrated membrane system impacted 8.45 × 10^−4^ DALY, 2.08 × 10^−6^ PDF species⋅year, and 16.80 USD2013. Figure 7 shows the relative contributions of the integrated membrane on human health, ecosystem quality, and resource availability. The hollow fiber membrane module had high characterization factors among the three impacts but was followed by the treated anaerobic POME contributing 41% and 46% to human health and ecosystems, respectively. The specific values in their respective units for the characterization factors of midpoint and endpoint categories are summarized in Tables S3 and S4. This finding contrasted with [32] which found that the inherent indirect human and freshwater toxicities linked to electro-oxidation outweigh the environmental benefits which were primarily owed to the production of electric energy required for the process unit operation and manufacturing of the anodes.

#### 3.2.3. Environmental Impacts between Processes of Integrated Membrane

**Figure 8:**
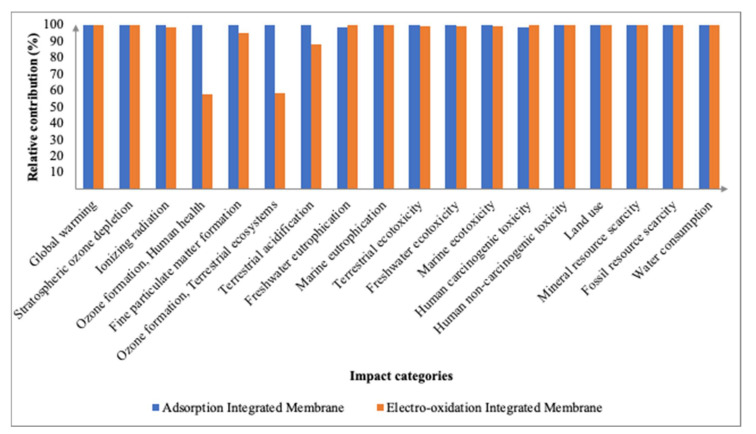
Relative contributions of midpoint impact categories between adsorption and electro-oxidation integrated membrane.

**Figure 9:**
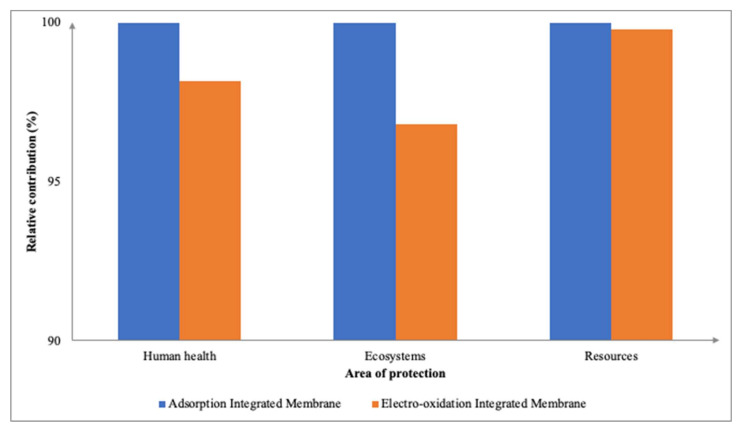
Relative contributions of endpoint impact categories between adsorption and electro-oxidation integrated membrane.

Utilizing the comparison feature in SimaPro, the midpoint and endpoint characterization factors between the two integrated membranes of adsorption and electro-oxidation were analyzed side-by-side. Figure 8 represents the midpoint characterization factors whilst Figure 9 represents the endpoint level damage assessment factors. This comparison should not be explicitly used as a defining factor upon which integrated membrane is determined as the best, as various other factors should be taken into consideration, and these will be further discussed in the next section. Regardless, this section aims to provide a brief overview of the overall impacts for both integrated membrane systems.

The adsorption integrated membrane contributed slightly higher (583.87 kg CO_2_-eq) in terms of GWP and the three endpoint characterization factors when compared to the electro-oxidation integrated membrane. The electro-oxidation integrated membrane contributed 582.93 kg CO_2_-eq toward GWP and had a minimal environmental impact on MEP at only 0.03 kg N-eq. The contrasting values are a result of different processes with different materials being employed in the integrated membrane system which may have been affected by the completeness of secondary data available for each process. This can be seen between the two integrated membrane processes of adsorption and electro-oxidation in which the inventory data for the electro-oxidation process provides less data on the materials used for the electro-oxidation treatment. This could explain the relative contribution of adsorption integrated membrane being higher than that of the electro-oxidation integrated membrane. This finding that the electro-oxidation integrated membrane is a more environmentally feasible technology is similar to the findings of [40] which observed that the electro-oxidation process produces fewer total environmental impacts, especially CO_2_ emissions, into the atmosphere. In addition, the electro-oxidation integrated membrane affected the resource’s availability the most at 16.80 USD2013, whilst affecting the ecosystem quality the least at 2.08 × 10^−6^ PDF species⋅year.

### 3.3. Environmental Impacts between Different Sources of Electricity

Three different scenarios with different sources of energy were analyzed to identify the impact of electricity sources on the environmental impact of the integrated membrane treatment. Given that the associated major contributor of these membrane treatment systems is caused by electricity consumption, strategies to improve this particular usage can be proposed to aid in making improvements. A previous study by [28] found that electricity consumption contributes the most to all life cycle impacts in which 73% is accounted to climate change, 80 % to terrestrial acidification, and 43 % to human toxicity. However, the FU defined for the study was 6 m^3^ of boiler feed grade treated water from aerobically digested POME compared to the present study which had an FU of 1 m^3^ of treated wastewater. Thus, this has an effect on the outcome of its environmental impacts due to the scale of the study.

The sources of electricity for this study were from the grid (baseline scenario), biogas energy and a combination of grid/solar electricity based on the Malaysian market named as Scenario 1, 2, and 3, respectively. As adsorption and electro-oxidation are different processes integrated into the membrane, the comparison is made between each integrated membrane when it is separated into different sections.

#### 3.3.1. Adsorption Integrated Membrane

**Figure 10:**
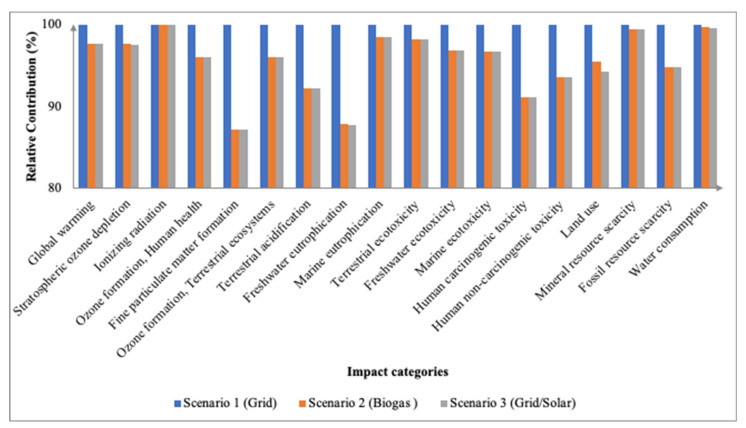
Relative contributions of midpoint impact categories between different sources of electricity for adsorption integrated membrane.

**Figure 11:**
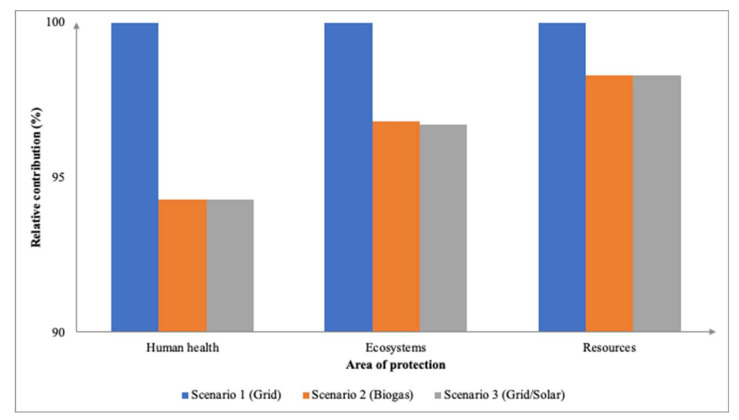
Relative contributions of endpoint impact categories between different sources of electricity for adsorption integrated membrane.

Across the 18 impact categories, the baseline scenario contributed the highest especially when compared against Scenario 2 and Scenario 3. The stark difference can be seen between the categories of PMFP (0.39 kg PM2.5 eq), FEP (0.07 kg P eq), and TAP (0.80 kg SO_2_ eq) where between the three scenarios, the grid impacted the environment the most followed by biogas and the solar/grid combination. This is in line with a comparative LCA study that showed renewable energy technologies have a much lower pollution-related environmental cost than coal-fired plants [41]. Moreover, non-renewable sources such as the grid are substantial contributors to human health, for instance, human respiratory problems are caused by power plant pollution. Figure 10 illustrates the midpoint impact categories based on their relative contribution. A study by [25] assessed the open lagoon technologies (COLT) which were combined with different membrane and composting technologies. The study found that the COLT biogas using membrane technology utilized the least amount of energy, 1 ton of fresh fruit bunches was defined as the FU for this LCA study. In respect of the endpoint approach (Figure 11), both Scenarios 2 and 3 had insignificant differences in terms of the three areas of protection with 8.11 × 10^−4^ DALY, 2.07 × 10^−6^ PDF species⋅yr, and 16.55 USD2013 affecting them. As for Scenario 1, the grid as a source of electricity impacted the highest with 8.61 × 10-^4^ DALY, 2.14 × 10^−6^ PDF species⋅yr, and 16.83 USD2013.

#### 3.3.2. Electro-Oxidation Integrated Membrane

**Figure 12:**
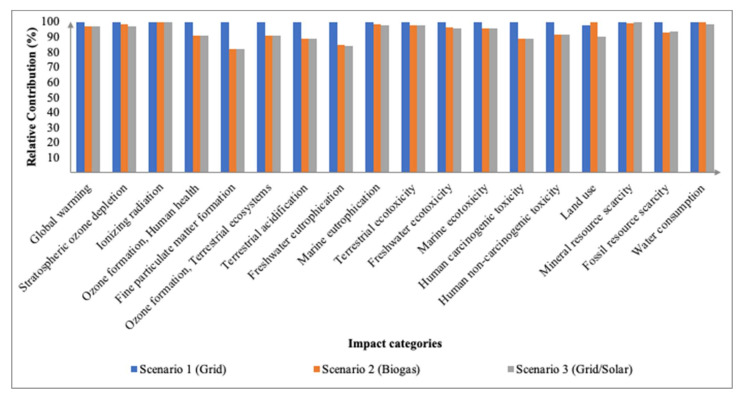
Relative contributions of midpoint impact categories between different sources of electricity for electro-oxidation integrated membrane.

**Figure 13:**
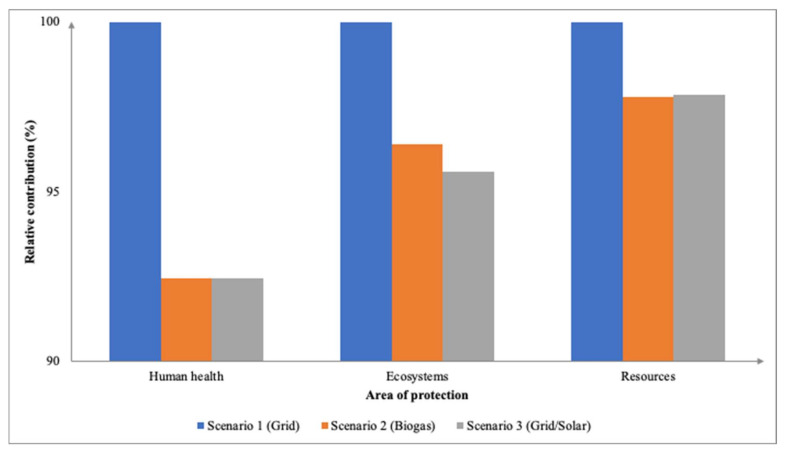
Relative contributions of endpoint impact categories between different sources of electricity for electro-oxidation integrated membrane.

It can be observed that throughout all the 18 impact categories (Figure 12), Scenario 1 had the highest environmental impact when compared with the other scenarios except for LUC and WCP in which Scenario 2 (biogas) had a higher impact at 19.50 m2a crop eq and 2.56 m^3^, respectively. Despite the fact that coal-fired plants are a major source of greenhouse gas (GHG) and hazardous air emissions, and the nuclear fuel cycle poses radiation risks due to fuel refining and disposal, renewable energy technology has social and environmental consequences, such as complex land-use change issues [42]. Nevertheless, the grid remains the major contributor across the categories especially towards PMFP (0.37 PM2.5 eq), FEP (0.07 kg P eq), and HTPc (7.80 kg 1,4-DCB). In terms of the endpoint areas of protection (Figure 13), akin to the adsorption integrated membrane, using the grid has the highest impact at 8.45 × 10^−4^ DALY, 2.08 × 10^−6^, and 16.80 USD2013.

## 4. Uncertainty Analysis

An uncertainty analysis was conducted using the Monte Carlo analysis method with a confidence interval of 95% for 1000 iterations. The uncertainty analysis was conducted for both the adsorption and electro-oxidation integrated membrane processes (Tables S5 and S6). The estimated values presented in Table 7 compare the coefficient of variation (CV) on the treatment of 1 m^3^ of wastewater with different sources of electricity. For the adsorption integrated membrane, the low CV for GWP, MEP, and HOFP shows that there is little uncertainty in the results obtained. The electro-oxidation integrated membrane had similar impact categories with low CV, with the only difference being the impact category of FFP instead of HOFP. However, both processes of the integrated membrane had WCP, IRP, and HTPc as their impact categories with higher CV that may originate from data variability issues from the database used, resulting in the highly uncertain results for the impact categories.

## 5. Challenges, Limitations, and Future Directions

One of the challenges in conducting this LCA study was the lack of data from previous studies concerning POME treatment, especially using an integrated membrane as a treatment system. Due to the lack of studies on LCA, secondary data for the life cycle inventory that is usually acquired by published data is scarce and data were not as complete as they could be. Besides that, the data utilized and obtained in this study were represented at a lab-scale where the inputs and environment are controlled and therefore they do not accurately portray its real-world impact. Due to this limitation, the two integrated membranes of adsorption and electro-oxidation had less significant differences in terms of their environmental impacts when studied between the processes. Regardless, comparing the two integrated adsorption and electro-oxidation membranes explicitly is not apt and instead their contributing factors should be noted as the processes employ different concepts of working principles, with electro-oxidation applying removal of organic/inorganic matter through reactions with hydroxyl radicals. There are also differences in the implementation of the method from the previous study based on the LCA framework. Most of the studies considered only the midpoint approach and defined their system boundary as a ‘gate-to-gate’ approach. A complete overall analysis of the POME membrane treatment system from ‘cradle-to-grave’ is still lacking and should be considered in the future to gain a more complete assessment of the impact on the environment. Environmental impacts and hotspots that represent real-world data can be achieved through upscaling the study by considering real-scale issues at the industrial level which would allow a more realistic view of the environmental impact. Upcoming LCA studies can also focus on analyzing scenarios from the treatment system with different types of energy sources, especially renewable energy, which could pave the road to a more environmentally friendly POME treatment, particularly in Malaysia.

## 6. Conclusions

The goal of this study was to conduct an LCA of two different integrated membranes methods to determine which is more environmentally friendly. It can be concluded from the in-depth ‘cradle-to-gate’ LCA that both the adsorption and the electro-oxidation integrated membrane treatment systems are mainly impacted by the production of a hollow fiber membrane module contributing at 42% to 99% at the midpoint level. At the endpoint level, 8.61 × 10^−4^ (adsorption integrated membrane) and 8.45 × 10^−4^ (electro-oxidation integrated membrane) DALY are the overall characterization factors of the endpoint categories that indicate the impact it has on human health. However, a direct comparison between the two integrated membrane systems is not suitable due to the differences in the process utilized and the thoroughness of the inventory data available. Additionally, in evaluating the impact of the electricity source, the grid/solar combination proves to have the lowest environmental impact between the treatment systems which is followed by biogas and then grid. Even so, the overall result of this study is influenced by the energy consumption based on Malaysia’s grid system that is highly dependent on coal production and may not represent the outcome of studies in other regions of the world. Future studies should focus on assessing the overall ‘cradle-to-grave’ or ‘cradle-to-cradle’ environmental impact of treating POME as well as different scenarios involving the energy usage of membrane treatment utilizing LCA.

## Figures and Tables

**Table 1:** Inventory data for palm nursery.

Input from Technosphere	Unit	Amount
*Materials and fuels*		
Seed	Seeds	15.42
Diesel (to run the pump for watering the seedlings)	L	0.01
Polybag	Kg	0.017
N Fertilizer, ammonium sulphate	Kg	0.05
N Fertilizer, urea	Kg	0.02
P (10%)	Kg	0.06
K (10%)	Kg	0.06
Cypermethrin	Kg	0.01
Dithane (a.i. mancozeb/dithiocarbamates)	Kg	0.02
Glufosinate ammonium	Kg	0.03
*Energy*		
Electricity	kWh	0.06
	MJ	2.64
*Transport*		
Van	Tkm	3.08 × 10^−4^
**Output to technosphere**		
*Products and co-products*		
Young oil palm	Palms	8
**Input from environment**		
*Resource*		
Water (river)	L	12

Source: [33].

**Table 2:** Inventory data for palm oil plantation.

Input from Technosphere	Unit	Amount
*Materials and fuels*		
Young oil palm	palms	8
Land	ha	0.05
Diesel	MJ	129.49
N Fertilizer, ammonium sulphate	kg	5.47
N Fertilizer, urea	kg	2.02
P Fertilizer	kg	4.49
K Fertilizer	kg	13.14
Cypermethrin	kg	8.20 × 10^−3^
Carbamate/Carbosulfan	kg	6.00 × 10^−3^
Carbamate/Carbofuran	kg	3.3 × 10^−3^
Metsulfuron metyl/Sulfonylurea	kg	4.50 × 10^−3^
Glufosinate ammonium	kg	0.05
Glyphosate	kg	0.04
Paraquat	kg	0.02
Chlorophacinone	kg	0.15
*Transport*		
Lorry 3 t	tkm	14.32
*Energy*		
Electricity	MJ	2.90 × 10^−3^
**Output to technosphere**		
*Products and co-products*		
Fresh fruit bunch	t	1.43
**Input from environment**		
*Resource*		
Water (Rainfall)	m^3^	1511.08

Source: [33].

**Table 3:** Inventory data for oil palm milling.

Input from Technosphere	Unit	Amount
*Materials and fuels*		
Fresh fruit bunch	t	1.43
Diesel	L	0.60
*Transport*		
Lorry 3t	tkm	14.32
*Energy*		
Power consumption from steam turbine	kWh	5.53
**Output to technosphere**		
*Products and co-products*		
Crude palm oil	t	0.29
Kernel	t	0.08
Shell	t	0.06
Mesocarp fibre	t	0.14
Empty bunch fibre	t	0.23
Palm oil mill effluent (POME)	t	1
**Input from environment**		
*Resource*		
Water for boiler and processing	m^3^	1.34
**Output to environment**		
*Emissions to air*		
Methane	kg	16.28
Carbon dioxide	kg	8.76
Boiler ash	kg	21.61

Source: [33].

**Table 4:** Inventory data for the pre-treatment stage.

Input from Technosphere	Unit	Amount
*Materials and fuels*		
POME	t	1
*Energy*		
Electricity from grid	kWh	0.02
**Output to technosphere**		
*Products and co-products*		
Anaerobic treated POME	m^3^	0.77
Biogas	m^3^	20.79
Electricity	kWh	0.03
Solid sludge	mt	0.11
**Input from environment**		
*Resource*		
Water	kg	11
**Output to environment**		
*Emissions to air*		
Methane	kg	8.94
Carbon dioxide	kg	14.79

Source: [33].

**Table 5:** Inventory data for adsorption process integrated membrane.

Input from Technosphere	Unit	Amount	Source
*Materials and fuels*			
Pretreated anaerobic POME	m^3^	0.77	[33]
Granular Activated Carbon production	kg	0.36	[30]
Hollow fiber membrane	*p*	1	
Chlorine	kg	6.00 × 10^−4^	[31]
Membrane cleaning agent (EDTA/NaOH)	kg	4.20 × 10^−3^	[31]
NaHCO_3_	kg	3.4 × 10^−3^	[31]
*Energy*			
Pumps	kWh	0.49	[31]
System cleaning (water heating)	kWh	4.4 × 10^−3^	[31]
Prefilter	kWh	0.04	[31]
**Output to Technosphere**	**Unit**	**Amount**	**Source**
*Products and co-products*			
Effluent treatment of POME	m^3^	1	
Spent catalyst management	kg	0.76	[30]
**Output to environment**			
*Emissions to air*			
Carbon dioxide, fossil	kg	2.40	[30]
Nitrogen dioxide	kg	0.28	[30]

**Table 6:** Inventory data for electro-oxidation process integrated membrane.

Input from Technosphere	Unit	Amount	Source
*Materials and fuels*			
Pretreated anaerobic POME	m^3^	0.77	[33]
Water storage	*p*	1.20 × 10^−7^	[32]
Steel, low-alloyed	kg	9.54 × 10^−3^	[32]
Chromium steel pipe	kg	1.76 × 10^−4^	[32]
Epoxy resin, liquid	kg	6.86 × 10^−4^	[32]
Water	m^3^	0.99	[32]
Hollow fiber membrane	*p*	1	
Chlorine	kg	6.00 × 10^−4^	[31]
Membrane cleaning agent (EDTA/NaOH)	kg	4.20 × 10^−3^	[31]
NaHCO_3_	kg	3.40 × 10^−3^	[31]
*Energy*			
Electricity, medium voltage	kWh	3.55	[32]
Pumps	kWh	0.49	[31]
System cleaning (water heating)	kWh	4.40 × 10^−3^	[31]
Prefilter	kWh	0.04	[31]
**Output to technosphere**			
*Products and co-products*			
Treated POME effluent	m^3^	1	

**Table 7:** Impacts of different sources of electricity of adsorption and electro-oxidation integrated membrane with uncertainty results (shown in terms of CV).

Impact Categories	Scenario 1: Grid (Baseline)		Scenario 2: Biogas		Scenario 3: Grid/Solar	
	Adsorption Integrated Membrane	Electro-Oxidation Integrated Membrane	Adsorption Integrated Membrane	Electro-Oxidation Integrated Membrane	Adsorption Integrated Membrane	Electro-Oxidation Integrated Membrane
PMFP (kg PM_2.5_-eq)	0.39 (9.89)	0.37 (10.38)	0.34	0.30	0.34	0.30
FFP (kg oil-eq)	57.84 (8.18)	58.10 (8.17)	54.82	54.16	54.82	54.19
FETP (kg 1,4-DCB)	8.97 (26.11)	8.90 (28.17)	8.69	8.55	8.69	8.54
FEP (kg P-eq)	0.07 (47.77)	0.07 (47.74)	0.06	0.06	0.06	0.06
GWP (kg CO_2_-eq)	583.87 (4.19)	582.93 (4.37)	570.15	565.08	570.15	565.12
HTPc (kg 1,4-DCB)	7.66 (127.22)	7.80 (102.41)	6.98	6.91	6.98	6.91
HTPnc (kg 1,4-DCB)	197.86 (30.47)	198.54 (33.45)	185.14	182.44	185.07	181.92
IRP (kBq Co−60-eq)	15.37 (135.40)	15.09 (122.35)	15.35	15.07	15.35	15.07
LUC (m^2^a crop-eq)	19.13 (26.62)	19.06 (25.64)	18.27	19.50	18.02	17.65
METP (kg 1,4-DCB)	11.88 (25.76)	11.80 (27.76)	11.49	11.32	11.49	11.30
MEP (kg N-eq)	0.03 (5.33)	0.03 (5.08)	0.03	0.03	0.03	0.03
SOP (kg Cu-eq)	0.73 (24.10)	0.73 (24.33)	0.73	0.72	0.73	0.73
HOFP (kg NO_x_-eq)	0.66 (5.34)	0.38 (9.50)	0.63	0.34	0.63	0.34
EOFP (kg NO_x_-eq)	0.67 (5.35)	0.39 (9.40)	0.64	0.35	0.64	0.35
ODP (kg CFC_11_-eq)	9.89 × 10^−5^ (13.10)	9.87 × 10^−5^ (12.97)	9.66 × 10^−5^	9.69 × 10^−5^	9.64 × 10^−5^	9.55 × 10^−5^
TAP (kg SO_2_-eq)	0.79 (10.93)	0.70 (12.59)	0.73	0.62	0.73	0.62
TETP (kg 1,4-DCB)	436.32 (40.72)	430.92 (42.75)	428.30	420.68	428.33	420.90
WCP (m^3^)	2.55 (1061.28)	2.54 (1233.73)	2.54	2.56	2.54	2.52

## Data Availability

Not applicable.

## Supplementary Materials

The following supporting information can be downloaded at: https://www.mdpi.com/article/10.3390/membranes12020246/s1, Table S1: Characterisation values of adsorption process integrated membrane at midpoint level; Table S2: Damage assessment values of adsorption process integrated membrane at endpoint level; Table S3: Characterisation values of electro-oxidation process integrated membrane at midpoint level; Table S4: Damage assessment values of electro-oxidation process integrated membrane at endpoint level; Table S5: Uncertainty analysis characterisation factors for adsorption-integrated membrane; Table S6: Uncertainty analysis characterisation factors for electro-oxidation integrated membrane.
