# Supplementary material for: Life Cycle Assessment of an Integrated Membrane Treatment System of Anaerobic-Treated Palm Oil Mill Effluent (POME)
Source: Membranes (Basel). 2022 Feb 21;12(2):246. doi: 10.3390/membranes12020246 (PMC8877097; doi:10.3390/membranes12020246)
Supplement: Supplementary file 1 [file membranes-12-00246-s001.zip › Supplementary .pdf]

Supplementary material

List of Tables

Table S1: Characterisation values of adsorption process integrated membrane at midpoint level.

Table S2: Damage assessment values of adsorption process integrated membrane at endpoint level.

Table S3: Characterisation values of electro-oxidation process integrated membrane at midpoint level.

Table S4: Damage assessment values of electro-oxidation process integrated membrane at endpoint level.

Table S5: Uncertainty analysis characterisation factors for adsorption-integrated membrane.

Table S6: Uncertainty analysis characterisation factors for electro-oxidation integrated membrane.

Table S1. Characterisation values of adsorption process integrated membrane at midpoint level.

Impact category	Unit	Total	Adsorption treated anaerobic POME	Pretreated Anaerobic POME	Activated carbon, granular {GLO}| market for activated carbon, granular | APOS, U	Sodium bicarbonate {GLO}| market for sodium bicarbonate | APOS, U	EDTA, ethylenediaminetetraacetic acid {GLO}| market for | APOS, U	Ultrafiltration module {GLO}| ultrafiltration module production, hollow fiber | APOS, U	Chlorine, liquid {RoW}| market for chlorine, liquid | APOS, U	Electricity, high voltage {MY}| electricity production, hard coal | APOS, U	Electricity, high voltage {MY}| electricity production, hard coal | APOS, U	Electricity, high voltage {MY}| electricity production, hard coal | APOS, U	Spent automobile catalyst {GLO}| market for | APOS, S
Global warming	kg CO2 eq	583.8737415	2.4	331.932112	1.21016041	0.00457764	0.01763618	246.029995	0.000637962	0.58587697	0.00526094	0.04184835	1.64563548
Stratospheric ozone depletion	kg CFC 11 eq	9.89013E-05	0	2.447E-06	2.9136E-07	1.5573E-09	7.1297E-09	9.5358E-05	6.42873E-10	1.0876E-07	9.7658E-10	7.7683E-09	6.7782E-07
Ionizing radiation	kBq Co-60 eq	15.36816348	0	0.02093818	0.04468263	0.00019839	0.00086251	15.0575024	5.11774E-05	0.00093059	8.3564E-06	6.6471E-05	0.24292278
Ozone formation, Human health	kg NOx eq	0.655531176	0.28	0.02540522	0.0027975	1.2693E-05	3.4291E-05	0.34275241	1.55881E-06	0.00112913	1.0139E-05	8.0652E-05	0.00330758
Fine particulate matter formation	kg PM2.5 eq	0.385918301	0.0308	0.04751614	0.00224739	1.2293E-05	2.4572E-05	0.2998002	1.44813E-06	0.00211185	1.8964E-05	0.00015085	0.0032346

Ozone formation, Terrestrial ecosystems	kg NOx eq	0.665968531	0.28	0.02550022	0.00282401	1.2854E-05	3.5592E-05	0.35303072	1.57321E-06	0.00113335	1.0177E-05	8.0954E-05	0.00333907
Terrestrial acidification	kg SO2 eq	0.794609808	0.1008	0.05929367	0.00585269	4.066E-05	5.7141E-05	0.61998292	2.37495E-06	0.0026353	2.3664E-05	0.00018824	0.00573315
Freshwater eutrophication	kg P eq	0.066630928	0	0.00828753	0.00050649	2.3003E-06	4.7495E-06	0.05653918	3.03643E-07	0.00036834	3.3075E-06	2.631E-05	0.00089242
Marine eutrophication	kg N eq	0.032611137	0	0.00054406	3.2943E-05	1.6046E-07	1.35E-05	0.03193421	3.09644E-08	2.4181E-05	2.1713E-07	1.7272E-06	6.0107E-05
Terrestrial ecotoxicity	kg 1,4-DCB	436.3164566	0	7.78153173	1.09186262	0.03968431	0.0592853	419.755916	0.00222832	0.34584899	0.00310558	0.0247035	7.21229034
Freshwater ecotoxicity	kg 1,4-DCB	8.968749871	0	0.27450485	0.02181044	0.00067368	0.00091461	8.51437395	4.04944E-05	0.01220033	0.00010955	0.00087145	0.14325051
Marine ecotoxicity	kg 1,4-DCB	11.88190433	0	0.38161352	0.02998564	0.00086855	0.00117691	11.2662536	5.25382E-05	0.01696075	0.0001523	0.00121148	0.18362902
Human carcinogenic toxicity	kg 1,4-DCB	7.66379871	0	0.66206999	0.03917139	0.00025116	0.00075338	6.86678445	2.87339E-05	0.0294256	0.00026423	0.00210183	0.06294795
Human non-carcinogenic toxicity	kg 1,4-DCB	197.8641758	0	12.2809097	0.93002878	0.01146186	0.01553772	181.583167	0.000798329	0.54582316	0.00490127	0.03898737	2.45256057
Land use	m2a crop eq	19.12660692	0	1.07060482	0.14270531	0.00155343	0.00182425	17.559122	0.000103291	0.04758287	0.00042727	0.00339878	0.29928486

Mineral resource scarcity	kg Cu eq	0.73039155	0	0.00482751	0.00056382	9.4943E-05	5.9503E-05	0.72130853	2.44737E-06	0.00021456	1.9266E-06	1.5326E-05	0.00330299
Fossil resource scarcity	kg oil eq	57.84471856	0	2.90546049	0.30728212	0.00096141	0.00675632	54.0974081	0.000156218	0.12913275	0.00115956	0.00922377	0.38717782
Water consumption	m3	2.549215272	0	0.02389905	0.00330096	0.00016186	0.00025038	2.50737444	1.50982E-05	0.00106219	9.538E-06	7.5871E-05	0.01306589

Table S2. Damage assessment values of adsorption process integrated membrane at endpoint level.

**Damage category**	**Unit**	**Total**	**Adsorption treated anaerobic POME**	**Pretreated Anaerobic POME**	**Activated carbon, granular {GLO}| market for activated carbon, granular | APOS, U**	**Sodium bicarbonate {GLO}| market for sodium bicarbonate | APOS, U**	**EDTA, ethylenediaminetetraacetic acid {GLO}| market for | APOS, U**	**Ultrafiltration module {GLO}| ultrafiltration module production, hollow fiber | APOS, U**	**Chlorine, liquid {RoW} | market for chlorine, liquid | APOS, U**	**Electricity, high voltage {MY}| electricity production, hard coal | APOS, U**	**Electricity, high voltage {MY}| electricity production, hard coal | APOS, U**	**Electricity, high voltage {MY}| electricity production, hard coal | APOS, U**	**Spent automobile catalyst {GLO}| market for | APOS, S**
Human health	DALY	0.000860579	0.000021858	0.00034342	2.8822E-06	1.5766E-08	3.8369E-08	0.00048576	1.81004E-09	2.0973E-06	1.8833E-08	1.498E-07	4.3436E-06
Ecosystems	PDF species.yr	2.14375E-06	6.42042E-08	9.6105E-07	6.6464E-09	4.1475E-11	8.9894E-11	1.0987E-06	3.84901E-12	3.0456E-09	2.7348E-11	2.1754E-10	9.7777E-09
Resources	USD2013	16.83327055	0	0.27881017	0.04903442	0.00021436	0.00227081	16.4095655	3.08489E-05	0.01239168	0.00011127	0.00088512	0.07995632

Table S3. Characterisation values of electro-oxidation process integrated membrane at midpoint level.

**Impact category**	**Unit**	**Total**	**Electro-oxidation treated anaerobic POME**	**Pretreated Anaerobic POME**	**Water storage {RoW} | construction | APOS, U**	**Epoxy resin, liquid {RER} | market for epoxy resin, liquid | APOS, U**	**Chromium steel pipe {GLO}| production | APOS, U**	**Steel, low-alloyed {GLO}| market for | APOS, U**	**Ultrafiltration module {GLO}| ultrafiltration module production, hollow fiber | APOS, U**	**Sodium bicarbonate {GLO} | market for sodium bicarbonate | APOS, U**	**EDTA, ethylenediaminetetraacetic acid {GLO}| market for | APOS, U**	**EDTA, ethylenediaminetetraacetic acid {GLO}| market for | APOS, U**	**Electricity, high voltage {MY}| electricity production, hard coal | APOS, U**	**Electricity, high voltage {MY}| electricity production, hard coal | APOS, U**	**Electricity, high voltage {MY}| electricity production, hard coal | APOS, U**	**Electricity, high voltage {MY}| electricity production, hard coal | APOS, U**
Global warming	kg CO_2 eq	582.931213	0	331.932112	0.0456407	0.0033036	0.00087177	0.01695061	246.029995	0.00457764	0.01763618	0.00251945	4.24461887	0.58587697	0.00526094	0.04184836
Stratospheric ozone depletion	kg CFC11 eq	9.8738E-05	0	2.447E-06	1.1408E-08	1.232E-09	2.7569E-10	5.0973E-09	9.5358E-05	1.5573E-09	7.1297E-09	1.0185E-09	7.8792E-07	1.0876E-07	9.7658E-10	7.7683E-09
Ionizing radiation	kBq Co-60 eq	15.0893681	0	0.02093818	0.00108932	0.00022117	3.7286E-05	0.00064813	15.0575024	0.00019839	0.00086251	0.00012322	0.00674206	0.00093059	8.3564E-06	6.6471E-05
Ozone formati	kg NO	0.37779922	0	0.02540522	0.00013739	7.1367E-06	2.3613E-06	4.2455E-05	0.34275241	1.2693E-05	3.4291E-05	4.8987E-06	0.00818044	0.00112913	1.0139E-05	8.0652E-05

on, Human health	x eq															
Fine particulate matter formation	kg PM2.5 eq	0.36505068	0	0.04751614	6.6145E-05	4.8828E-06	3.118E-06	3.8047E-05	0.2998002	1.2293E-05	2.4572E-05	3.5103E-06	0.01530012	0.00211185	1.8964E-05	0.00015085
Ozone formation, Terrestrial ecosystems	kg NOx eq	0.38821559	0	0.02550022	0.00014122	7.6342E-06	2.4146E-06	4.434E-05	0.35303072	1.2854E-05	3.5592E-05	5.0846E-06	0.00821103	0.00113335	1.0177E-05	8.0954E-05
Terrestrial acidification	kg SO_2 eq	0.70151002	0	0.05929367	0.00012034	9.9547E-06	3.5929E-06	5.3916E-05	0.61998292	4.066E-05	5.7141E-05	8.1629E-06	0.01909247	0.0026353	2.3664E-05	0.00018824
Freshwater eutrophication	kg P eq	0.06792491	0	0.00828753	1.2022E-05	1.0802E-06	3.2707E-07	1.0528E-05	0.05653918	2.3003E-06	4.7495E-06	6.785E-07	0.00266857	0.00036834	3.3075E-06	2.631E-05
Marine eutrophication	kg N eq	0.03269762	0	0.00054406	1.2636E-06	8.4056E-08	3.1013E-08	1.0672E-06	0.03193421	1.6046E-07	1.35E-05	1.9285E-06	0.00017519	2.4181E-05	2.1713E-07	1.7272E-06
Terrestrial ecotoxicity	kg 1,4-DCB	430.916807	0	7.78153175	0.1986771	0.01114175	0.02558744	0.15721642	419.755915	0.03968431	0.0592853	0.00846933	2.50564066	0.34584899	0.00310558	0.0247035
Freshwater	kg 1,4-	8.89894145	0	0.27450485	0.00251844	0.00018237	6.5962E-05	0.00400549	8.51437393	0.00067368	0.00091461	0.00013066	0.08839012	0.01220033	0.00010955	0.00087145

ecotoxicity	DCB																
Marine ecotoxicity	kg 1,4-DCB	11.8000305	0	0.38161353	0.00331443	0.00023069	9.4254E-05	0.00510691	11.2662536	0.00086855	0.00117691	0.00016813	0.12287894	0.01696075	0.0001523	0.00121148
Human carcinogenic toxicity	kg 1,4-DCB	7.79522122	0	0.66206999	0.00739007	0.00011353	0.00035426	0.01241964	6.86678444	0.00025116	0.00075338	0.00010763	0.21318547	0.0294256	0.00026423	0.00210183
Human non-carcinogenic toxicity	kg 1,4-DCB	198.535733	0	12.2809097	0.04519018	0.00319282	0.00125989	0.0486498	181.583167	0.01146186	0.01553772	0.00221967	3.95443309	0.54582316	0.00490127	0.03898737
Land use	m2a crop eq	19.0589316	0	1.07060482	0.02390684	0.00040965	0.00020632	0.00490169	17.5591221	0.00155343	0.00182425	0.00026061	0.34473302	0.04758287	0.00042727	0.00339878
Mineral resource scarcity	kg Cu eq	0.72992956	0	0.00482751	0.000942	1.1315E-05	8.8826E-05	0.00080218	0.72130853	9.4943E-05	5.9503E-05	8.5004E-06	0.00155445	0.00021456	1.9266E-06	1.5326E-05
Fossil resource scarcity	kg oil eq	58.0997194	0	2.90546049	0.00827338	0.00129343	0.00018913	0.00334221	54.0974081	0.00096141	0.00675632	0.00096519	0.93555359	0.12913275	0.00115956	0.00922377
Water consumption	m3	2.54117427	0	0.02389905	0.00043502	4.3468E-05	7.1589E-06	0.00012406	2.50737444	0.00016186	0.00025038	3.5769E-05	0.00769545	0.00106219	9.538E-06	7.5871E-05

Table S4. Damage assessment values of electro-oxidation process integrated membrane at endpoint level.

Damage category	Unit	Total	Electro-oxidation treated anaerobic POME	Pretreated Anaerobic POME	Water storage {RoW} | construction | APOS, U	Epoxy resin, liquid {RER}| market for epoxy resin, liquid | APOS, U	Chromium steel pipe {GLO}| production | APOS, U	Steel, low-alloyed {GLO}| market for | APOS, U	Ultrafiltration module {GLO}| ultrafiltration module production, hollow fiber | APOS, U	Sodium bicarbonate {GLO} | market for sodium bicarbonate | APOS, U	EDTA, ethylenediaminetetraacetic acid {GLO}| market for | APOS, U	EDTA, ethylenediaminetetraacetic acid {GLO}| market for | APOS, U	Electricity, high voltage {MY}| electricity production, hard coal | APOS, U	Electricity, high voltage {MY}| electricity production, hard coal | APOS, U	Electricity, high voltage {MY}| electricity production, hard coal | APOS, U	Electricity, high voltage {MY}| electricity production, hard coal | APOS, U
Human health	DALY	0.00084472	-2.1978E-06	0.00034342	1.1944E-07	7.3266E-09	4.2451E-09	9.2026E-08	0.00048576	1.5766E-08	3.8369E-08	5.4813E-09	1.5194E-05	2.0973E-06	1.8833E-08	1.498E-07
Ecosystems	PDF species.yr	2.0754E-06	-1.0375E-08	9.6105E-07	3.9995E-10	1.747E-11	5.9789E-12	1.2083E-10	1.0987E-06	4.1475E-11	8.9894E-11	1.2842E-11	2.2065E-08	3.0456E-09	2.7348E-11	2.1754E-10
Resources	USD 2013	16.7983461	0	0.27881017	0.00263423	0.0004274	6.15E-05	0.00087315	16.4095656	0.00021436	0.00227081	0.0003244	0.08977642	0.01239168	0.00011127	0.00088512

Table S7. Uncertainty analysis characterisation factors for adsorption-integrated membrane.

Impact category	Unit	Mean	Median	SD	CV	2.5%	97.5%	SEM
Global warming	kg CO2 eq	583.367871	580.917583	24.4477779	4.19079951	543.023201	636.972507	0.77310662
Stratospheric ozone depletion	kg CFC11 eq	9.8293E-05	9.7182E-05	1.2871E-05	13.095062	7.7052E-05	0.00012678	4.0703E-07
Ionizing radiation	kBq Co-60 eq	15.8233735	8.68691082	21.4248999	135.40033	2.0730122	72.8253376	0.67751482
Ozone formation, Human health	kg NOx eq	0.65335502	0.65012081	0.03488505	5.33937116	0.59484801	0.72965731	0.00110316
Fine particulate matter formation	kg PM2.5 eq	0.38399881	0.37896091	0.03794499	9.88153978	0.32462102	0.46810891	0.00119993
Ozone formation, Terrestrial ecosystems	kg NOx eq	0.66378212	0.66066092	0.03552691	5.352195	0.60438042	0.74258494	0.00112346
Terrestrial acidification	kg SO2 eq	0.79028208	0.77746676	0.0863845	10.9308436	0.65719435	0.99072199	0.00273172
Freshwater eutrophication	kg P eq	0.06509487	0.05813612	0.03109714	47.7720385	0.02935041	0.14693006	0.00098338
Freshwater ecotoxicity	kg 1,4-DCB	8.8672656	8.45634995	2.31525358	26.1101187	5.77177313	14.7899254	0.07321475
Marine eutrophication	kg N eq	0.03252974	0.03247775	0.00173231	5.32530788	0.02921748	0.03617762	5.478E-05
Terrestrial ecotoxicity	kg 1,4-DCB	428.661938	390.387888	174.545477	40.7186787	259.063315	804.222208	5.51961263
Human carcinogenic toxicity	kg 1,4-DCB	8.02472254	6.06037226	10.2088543	127.217537	3.74528413	23.9243776	0.32283232
Human non-carcinogenic toxicity	kg 1,4-DCB	193.46277	180.746893	58.9425631	30.4671348	122.500603	341.479359	1.8639275
Land use	m2a crop eq	19.1000329	18.1590954	5.0848691	26.6223055	11.7863771	31.239945	0.16079768
Marine ecotoxicity	kg 1,4-DCB	11.7467508	11.2112855	3.02589874	25.7594529	7.63622452	19.7273611	0.09568732
Mineral resource scarcity	kg Cu eq	0.72150046	0.68965984	0.17388995	24.1011557	0.46835566	1.15428693	0.00549888
Fossil resource scarcity	kg oil eq	57.6514577	57.2813474	4.71614178	8.18043806	49.7326259	68.2792121	0.1491375
Water consumption	m3	3.35032523	5.98167479	35.5564632	1061.28393	-70.70422	64.830888	1.12439409

Table S8. Uncertainty analysis characterisation factors for electro-oxidation integrated membrane.

Impact category	Unit	Mean	Median	SD	CV	2.5%	97.5%	SEM
Global warming	kg CO2 eq	584.714947	581.773908	25.5413755	4.36817558	544.510098	644.704718	0.80768921
Stratospheric ozone depletion	kg CFC11 eq	9.9069E-05	9.7814E-05	1.285E-05	12.9704619	7.6656E-05	0.00012652	4.0634E-07
Ionizing radiation	kBq Co-60 eq	15.8720402	8.77767541	19.4189313	122.346787	1.72529522	75.7273386	0.61408053
Ozone formation, Human health	kg NOx eq	0.37922882	0.37546649	0.03601312	9.49640826	0.31929002	0.45966541	0.00113883
Fine particulate matter formation	kg PM2.5 eq	0.36684392	0.3632343	0.03808109	10.3807336	0.30129128	0.45927495	0.00120423
Ozone formation, Terrestrial ecosystems	kg NOx eq	0.38968661	0.38563692	0.03665785	9.40700762	0.32883326	0.47158419	0.00115922
Terrestrial acidification	kg SO2 eq	0.70524795	0.69402731	0.08878963	12.5898458	0.55889167	0.92766633	0.00280777
Freshwater eutrophication	kg P eq	0.06782316	0.06106219	0.03237621	47.7362059	0.0270261	0.15528067	0.00102383
Marine eutrophication	kg N eq	0.03275906	0.0327097	0.00166294	5.07626926	0.02961166	0.03599552	5.2587E-05
Terrestrial ecotoxicity	kg 1,4-DCB	440.785099	392.792925	188.433244	42.7494588	256.073768	936.687305	5.95878239
Freshwater ecotoxicity	kg 1,4-DCB	9.0035229	8.45925312	2.53665983	28.174081	5.52453187	15.5226611	0.08021623
Marine ecotoxicity	kg 1,4-DCB	11.9303953	11.246475	3.31142155	27.7561763	7.3800956	20.5527245	0.10471634
Human carcinogenic toxicity	kg 1,4-DCB	7.85436418	6.10742792	8.04336819	102.406357	3.82596706	21.9892197	0.25435364
Human non-carcinogenic toxicity	kg 1,4-DCB	199.436202	184.780073	66.7063955	33.4474858	120.946063	360.93625	2.10944144
Land use	m2a crop eq	19.1828331	18.4041192	4.91851495	25.6401905	12.1267303	30.450068	0.1555371
Mineral resource scarcity	kg Cu eq	0.74213165	0.71407882	0.1805971	24.3349146	0.46303946	1.17062387	0.00571098
Fossil resource scarcity	kg oil eq	58.5141493	58.2226253	4.78051177	8.16983897	50.2578551	69.4762084	0.15117306
Water consumption	m3	2.84535782	4.45484355	35.1041246	1233.73322	-67.863748	73.2522273	1.11008989
